# Vimentin 3, the New Hope, Differentiating RCC versus Oncocytoma

**DOI:** 10.1155/2015/368534

**Published:** 2015-04-07

**Authors:** Melanie von Brandenstein, Katharina Puetz, Monika Schlosser, Heike Löser, Joachim P. Kallinowski, Daniel Gödde, Reinhard Buettner, Stefan Störkel, Jochen W. U. Fries

**Affiliations:** ^1^Institute of Pathology, University Hospital of Cologne, Kerpenerstraße 62, 50924 Cologne, Germany; ^2^Institute of General, Visceral and Minimal Invasive Surgery, Clinic Northwest, Steinbacher Hohl 2-26, 60488 Frankfurt am Main, Germany; ^3^Institute of Pathology, Helios Clinic Wuppertal, University Clinic Witten-Herdecke, Heusnerstraße 40, 42283 Wuppertal, Germany

## Abstract

Vimentin is currently used to differentiate between malignant renal carcinomas and benign oncocytomas. Recent reports showing Vimentin positive oncocytomas seriously question the validity of this present diagnostic approach. Vimentin 3 is a spliced variant and ends with a unique C-terminal ending after exon 7 which differentiates it from the full length version that has 9 exons. Therefore, the protein size is different; the full length Vimentin version has a protein size of ~57 kDa and the truncated version of ~47 kDa. We designed an antibody, called Vim3, against the unique C-terminal ending of the Vimentin 3 variant. Using immune histology, immune fluorescence, Western blot, and qRT-PCR analysis, a Vim3 overexpression was detectable exclusively in oncocytoma, making the detection of Vim3 a potential specific marker for benign kidney tumors. This antibody is the first to clearly differentiate benign oncocytoma and the mimicking eosinophilic variants of the RCCs. This differentiation between malignant and benign RCCs is essential for operative planning, follow-up therapy, and patients' survival. In the future the usage of Vimentin antibodies in routine pathology has to be applied with care. Consideration must be given to Vimentin specific binding epitopes otherwise a misdiagnosis of the patients' tumor samples may result.

## 1. Introduction

An oncocyte is an epithelial cell characterized by an excessive amount of mitochondria. Hamperl named them in 1931 after the Greek word “onkousthai” (to swell) and first described them as a distinct cell system consisting of large epithelial cells with irregular nuclei and finely granular, acidophilic cytoplasm [1]. The fundamental morphological nature of oncocytes, an abundance of mitochondria, was firmly established by electron microscopy [2]. Since then oncocytes have been detected in various organs (i.e., thyroid, parathyroid, and salivary glands) as well as in different tumors (i.e., oncocytomas, Hürthle cell tumors of the thyroid, oxyphilic adenoma of parathyroid gland, and Warthin's tumor of salivary gland) (encyclopedia of Biol Chem 2004).

Renal oncocytomas, initially identified by Zippel, in 1942 [3], have been regarded as predominantly benign renal neoplasms since the first study by Klein and Valensi [4], although occasional reports of malignant cases have been reported [5]. The major diagnostic problem is the differential to other renal tumors: (i) the eosinophilic or granular variant of clear cell renal carcinoma (RCC) and (ii) the chromophobe RCC. Differential diagnosis currently uses immune histology to differentiate malignant renal cell carcinoma from oncocytoma. For chromophobe carcinoma, positivity for claudin 8 and negativity for claudin 7 have been shown as the characteristic constellation [6]. To differentiate the chromophobe and eosinophilic RCC from oncocytoma, the positivity for Vimentin, a structural protein, has been used to identify the former [7]. However, a series of oncocytomas has recently been reported in which a Vimentin positivity has been observed, making the differentiation questionable, particularly in preoperative evaluation [8]. Hes et al. analyzed 234 oncocytoma of which 73% were positive for Vimentin staining [8]. Vimentin is an intermediate-sized filament that functions in cellular signal transduction, structural integrity of cells and tissues, and adhesion and migration [9]. In 2007 a spliced variant of Vimentin with a unique C-terminal ending was detected by a working group at the Craig Venter Institute (NHLBI Resequencing and Genotyping Service (RSG), N01-NV-48196, J. Craig Venter Institute, Rockville, MD 20850) and published online in PubMed (Accession number ACA06103.1). In 2011 Thakkar et al. [10] described the presence of this variant in gliomas. However, no further analysis or investigation regarding its role has been performed.

Based on the knowledge that the spliced variant of Vimentin is 35 amino acids smaller than the full length variant, we compared both sequences with the detailed information of the Vimentin 3B4 antibody. From the literature it is known that the 3B4 Vimentin antibody detects the rod domain [11] which is a homologue to the truncated Vimentin variant 3 (Vim3) rod domain. Thus, it seemed possible that the protein expression of Vimentin described in the literature by immune histology resulted from the combined detection not only of the protein from full length, but also of the spliced variant of Vimentin, namely, Vim3.

**Figure 1:**
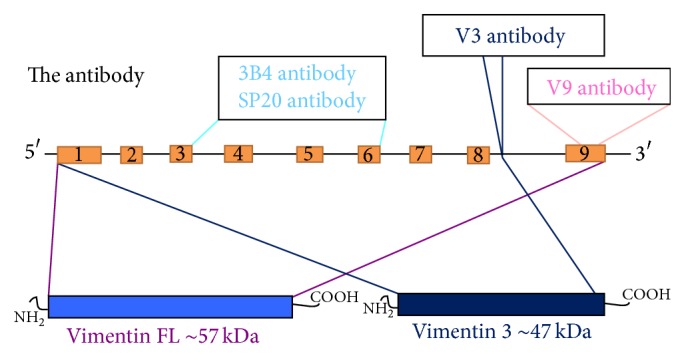
Differential location of the different antibodies against Vimentin. The commercially available antibodies of the clones 3B4 and SP20 were against the rod domain (slight blue); the antibody clone V9 is against C-terminal ending of the full length variant (slight purple); and the truncated antibody against the unique C-terminal ending Vim3 is indicated with dark blue.

Most of the commercially available antibodies (clones 3B4 and SP20) are against epitopes located in the rod domain of Vimentin (Figure 1). The clone V9 is directed against the tail-domain of Vimentin. However, for the detection of the truncated Vimentin variant 3 (Vim3), the Vim3 antibody is used, which is designed against the unique C-terminal ending.

In case of renal tumors with eosinophilic apperance, which mimic oncocytoma, the differential diagnosis between RCCs and oncocytomas is based on a panel of different antibodies. In particular the presence or absence of Vimentin staining of paraffinized tumor samples can be of great importance for the differentiation between malignant and benign tumors.

This diagnostic approach has to be reevaluated, since a spliced Vimentin isoform exists. This is also detectable with the currently used antibodies against the N-terminal sequence; thus Vimentin positivity is no longer a diagnostic feature per se of malignant RCCs. Thus, in this paper we analyzed the presence of Vim3 versus the full length Vimentin in RCCs, especially the eosinophilic variant of RCCs versus oncocytomas.

We designed primers which detect either the full length version of Vimentin or its spliced variant, Vim3. After performing a qRT-PCR on paraffin embedded tissues of the different RCCs and oncocytoma we could indeed show that Vim3 is the predominant variant in oncocytoma. Furthermore, we designed an antibody exclusively detecting the unique C-terminus of Vim3.

This is the first report describing the presence and the structural differences of Vim3 versus the full length Vimentin. Our data present strong evidence that Vim3 is the isoform responsible for the so-called Vimentin positive oncocytomas described in the literature. Furthermore, we show that the V9 Vimentin antibody as well as antibodies detecting the full length version of Vimentin* cannot* be used any longer for differential diagnosis between RCCs and oncocytomas, because these result in misdiagnoses with potentially grave consequences for the patients involved.

## 2. Materials and Methods

### 2.1. Antibody Design and Quantification

The Vim3 antibody was commercially designed (EZbiolab, Inc.) using the unique C-terminal ending of Vim3 as target (for detailed information please see patent by University of Cologne, Brandenstein/Fries, patent number EP 13160876.2-1405). The Vim3 expression versus that of full length Vimentin (clone V9) (Santa Cruz, Heidelberg) was shown using immune histology on paraffin embedded colon mucosa biopsies from our pathology archive. Western blot analysis (see below) of macrodissected material of cryptal epithelial cells and lymphoid cells was performed for further evaluation and proof of specificity of the newly designed antibody.

### 2.2. Immune Fluorescence of Paraffin Embedded Tissues

4 *μ*m thick paraffin embedded tissue sections were deparaffinized by incubation for 1 x 10 min in xylene, followed by 1 x 5 min 100% ethanol and 1 min 70% ethanol, and then rinsed with distilled water. The slides were digested with Proteinase K for 30 min at room temperature. After an incubation period in 5% PBS milk for 30 min, the slides were reincubated for 1 hour at room temperature with specific primary antibodies (Vim3) in 3% PBS milk. Following washes with PBS, the sections were incubated with a secondary FITC-anti-rabbit antibody (Santa Cruz). Subsequent to rinsing with PBS, the slides were then counterstained with DAPI mounting medium (nuclear staining) and cover slipped.

### 2.3. Immune Histology of Paraffin Embedded Tissues

Paraffin embedded tissue sections (4 *μ*m thick) were deparaffinized by incubation for 2–5 minutes in xylene, followed by 2-3 minutes in 100% ethanol and 1 minute in 95% ethanol, and then rinsed with distilled water. The slides were incubated with a specific serum blocker (anti-rabbit) for 30 minutes in order to avoid nonspecific binding. After that incubation period, the slides were reincubated for 1 hour at room temperature with specific primary antibodies (Vim3, EZBiolab, Inc. Carmel, USA, Vimentin V9, Santa Cruz, Heidelberg, Germany, AMACR and CD117 [12], Dako, Hamburg, Germany). Following washes with PBS–Tween 20, the sections were incubated with a secondary, anti-rabbit antibody (Santa Cruz, Heidelberg, Germany). After rinsing with PBS–Tween 20, the slides were reincubated for 2 minutes in 95% ethanol, followed by 2-3 minutes in 100% methanol, counterstained with H&E, and cover slipped.

The analyzed paraffin embedded tissue sections were from retrospective nephrectomies; nevertheless we performed a blind study, so any bias of the results could be excluded.

### 2.4. Oncocytic Tumors

Since human materials were used, procedures were followed as outlined in accordance with ethical standards formulated in the Declaration of Helsinki 1975, with preapproval by the Ethics Committee at the University Hospital, Cologne, Germany (reference number 09-232).

### 2.5. Quantitative Real-Time PCR (qRT-PCR)

The qRT-PCR was performed as previously described [13, 14].

For quantitative analysis, *β*-actin was measured. All samples were normalized to *β*-actin as the reference gene. All experiments were performed in triplicate. Relative fluorescence was calculated using the ΔΔ-CT method, as outlined in User Bulletin 2 (PE Applied Biosystems, Darmstadt, Germany). The statistical significance of qPCR values at different time points was assessed by Student's paired *t*-test.  Table 2 provides primer information.

### 2.6. RNA-Extraction Paraffin Embedded Tissues and RT-PCR

Formalin-fixed and paraffinized (FFPE) human tissue samples, from the archives of the Department of Pathology, University Hospital of Cologne, Cologne, Germany, and the Department of Pathology, Helios Clinic Wuppertal, University Clinic Witten-Herdecke, Wuppertal, Germany, were used.

RNA extraction from FFPE tissue was performed according to the RNeasy FFPE kit (Qiagen, Germany). RNA quantification was accomplished using NanoDrop technology.

The cDNA was obtained from 250 ng of RNA using random primers and SuperScript III reverse transcriptase, according to the manufacturer's protocol (Invitrogen, Darmstadt, Germany).

### 2.7. Statistical Analysis

For statistical analysis the GraphPad Prism 5 program was used. Analysis of variance (ANOVA) was performed and the significant differences were calculated and indicated by stars (^∗^ *P* < 0.05, ^∗∗^ *P* < 0.01, and ^∗∗∗^ *P* < 0.001). All differences without indication were not statistically significant.

### 2.8. Western Blot

All Western blots were performed in triplicate as outlined in detail before (Gerstung et al. [13]). *β*-actin served as loading control (Santa Cruz, Heidelberg, Germany). The Vimentin 3 antibody was used in a 1 : 500 dilution, and the V9 antibody (Santa Cruz) against full length Vimentin was employed in 1 : 1000 as recommended by the supplier. Protein extraction from paraffinized tissue was done as described in Ikeda et al. [15]. The 4 *μ*m paraffinized tissue samples were incubated in Xylol for 15 sec, mixed, and then centrifuged for 2 min at full speed and at room temperature. 100% ethanol was added to the pellet for 2 min, then mixed, and again centrifuged for 2 min at full speed and at room temperature. After carefully discarding the supernate, the pellet was air dried. 50 *μ*L of RIPA buffer was added, incubated at 100°C for 20 min, and then followed by an incubation period of 2 hours at 60°C. The samples were subsequently centrifuged at full speed at 4°C for 20 min. The supernate was then stored at −80°C until further use. Protein quantification was performed as previously described [13].

## 3. Results

### 3.1. Antibody Evaluation

**Figure 2:**
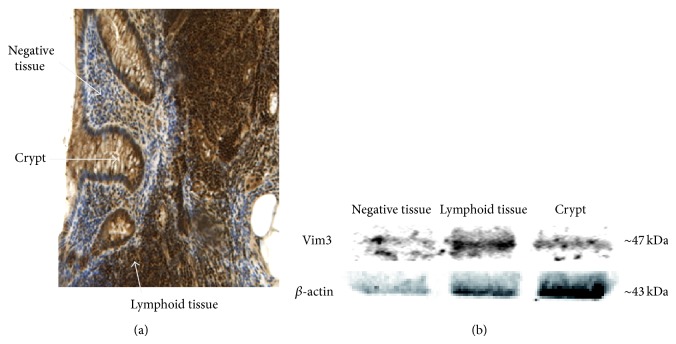
Evaluation of Vim3 antibody. (a) Immune histology showing expression of Vim3 in colonic crypt epithelium and in lymphocytes. (b) Western blot analysis after macrodissection of crypt epithelium and lymphocytes. *β*-actin serves as loading control.

Since Vimentin is commonly known primarily as a mesenchymal marker, we characterized the Vim3 antibody using frozen sections of appendiceal tissue containing epithelial, mesenchymal, and lymphatic tissue elements. As Figure 2 shows, Vim3 was expressed in colonic crypt epithelium, particularly in the regeneratively active part of the crypt, in mesenchymal cells, and in lymphocytes. A Western blot was performed to verify the expected size of the Vim3 splice form being 47 kDa (Figure 2), while the full length molecule was predictably 57 kDa (data not shown).

We also established the Vim3 antibody binding pattern in renal tissues. The Vimentin full length molecule was evident in different types of mesenchymal cells (such as fibroblasts and smooth muscle cells) and also in proximal tubule cells.

### 3.2. mRNA Detection of Vimentin and Vim3

The full length molecule of Vimentin is used as a marker to differentiate benign oncocytomas, expected to be negative, from malignant renal cell carcinomas being Vimentin positive. Our qRT-PCR evaluation of renal tumors confirmed this finding in cases from the pathology archives, while demonstrating that full length Vim3 was expressed in (Table 1) Onco (Oncocytoma). RCC subtypes express lower levels of Vim3 mRNA.

### 3.3. Protein Detection of Vim3 versus Full Length Vimentin in RCCs

By immune histology on paraffinized tissue slices from renal tumors, full length Vimentin protein was found to be strongly expressed in clear cell RCCs and papillary RCCs. Chromophobe RCCs showed a weak reactivity with the antibody, while oncocytomas demonstrated no reactivity. In contrast, Vim3 expression was strong in oncocytomas, while all three malignant RCCs subtypes were negative (Figure 4).

Consequently, using immune fluorescence analyses of the different RCC subtypes and the oncocytoma a clear expression of Vim3 was only detectable in oncocytoma. The oncocytoma mimicking variant of RCC, namely, the eosinophilic variant, was negative for Vim3 (Figure 5).

### 3.4. Collision Tumor

To further demonstrate the applicability of the new Vim3 antibody, a collision tumor consisting of two different tumor subtypes was used.  Figure 6 shows the H&E staining of its papillary RCC differentiation. Since the patient suffered from pleural metastases, it was important to identify their origin. Therefore, we performed an immune fluorescence staining for Vimentin FL positive (V9) and for Vim3. This indicated that the first tumor type with V9 being positive and Vim3 being negative was the malignant component. The histogenesis of the second tumor type found in the tissue sample was questionable, possibly being a “real” oncocytoma. After immune fluorescence for Vimentin FL (V9) as well as for Vim3 was performed, only the Vim3 staining showed positive areas, indicating that the second tumor type was indeed an oncocytoma.

## 4. Discussion

In this paper, we characterize a Vimentin splice isoform, called Vimentin 3 (Vim3), as a potentially important structural cellular protein. Its unique structure leads to a 10 kDa smaller protein (Figure 1), which is more widely expressed than its full length counterpart, particularly in epithelial cells and lymphocytes (Figure 2).

**Figure 3:**
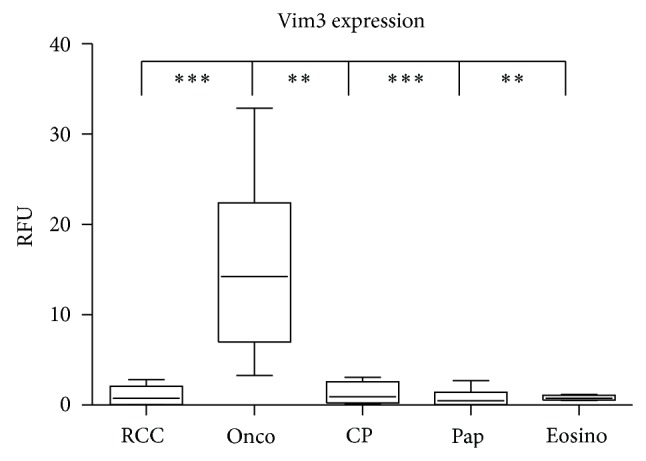
qRT-PCR analysis for Vim3 in oncocytoma versus clear cell renal cell carcinoma (RCC), chromophobe RCC (CP), papillary RCC (Pap), and eosinophilic RCC (Eosino). *β*-actin was used as reference gene. ^∗^ *P* < 0.05; ^∗∗^ *P* < 0.01; ^∗∗∗^ *P* < 0.001, and all differences without indication are not statistically significant.

To study the importance of Vim3 for renal tubule cells further, we analyzed Vim3 versus full length Vimentin expression by qRT-PCR in renal tumors (Figure 3). Surprisingly, while RCCs have high amounts of transcribed full length Vimentin, they are almost Vim3 negative. In contrast, the reverse is true for oncocytomas: while their negativity for full length Vimentin is not surprising (and being a criteria for their identification), the levels for Vim3 are unexpectedly high. The papillary RCC subtype (Pap) has small mRNA level of Vimentin full length and Vim3 detectable by qRT-PCR.

**Figure 4:**
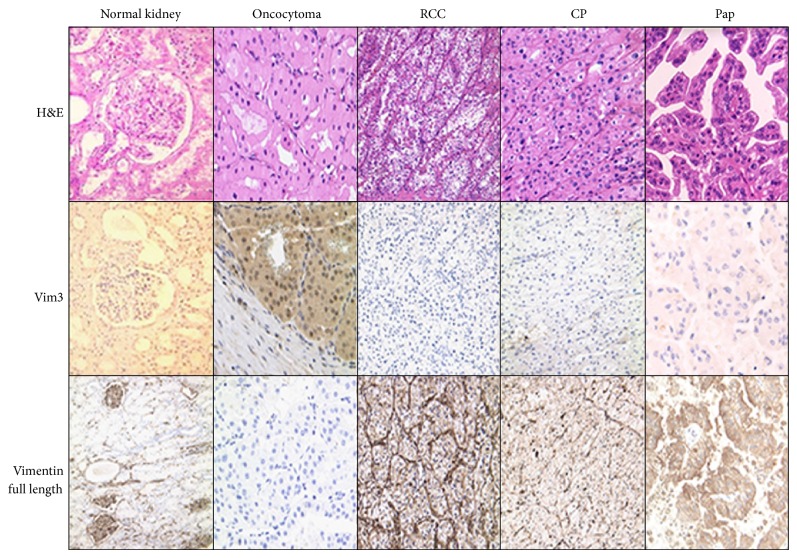
Immune histological analysis of the expression pattern between oncocytoma and RCC subtypes. Full length Vimentin positive tumor cells are observed in clear cell and papillary RCCs, while Vim3 positive cells are only found in oncocytoma, which otherwise are negative for full length Vimentin. H&E staining of typical tumor morphology.

Nevertheless, due to some posttranscriptional modifications, the Vim3 signal is not detectable by immune histology (Figure 4), while a strong Vimentin (full length) staining can be easily detected. The only positive signal, regarding the Vim3, was detectable in case of oncocytoma, and the “normal” tissue section was negative for Vim3 (Figure 4).

**Figure 5:**
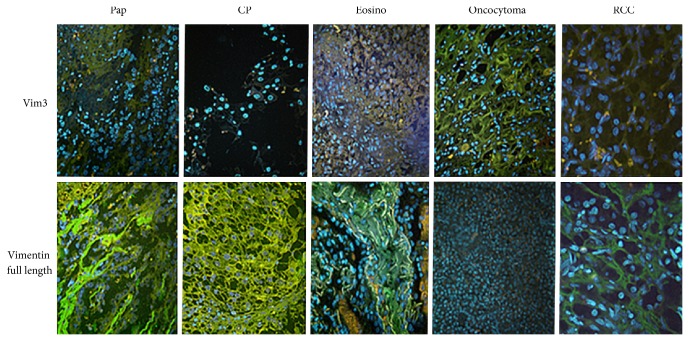
Immune fluorescence of oncocytoma and RCC subtypes. Full length Vimentin positive tumor cells are observed in clear cell and papillary RCCs, while Vim3 positive cells are only found in oncocytoma, which otherwise are negative for full length Vimentin. H&E staining of typical tumor morphology; original magnification ×400.

This result as well as the immune fluorescence results (Figure 5) identifies Vim3 as potential immune histology marker for renal oncocytomas.

Currently, it is still common practice in routine pathology to differentiate renal cell carcinomas from carcinomas of histogenetically different origins by using immune histology with cytokeratins and Vimentin. In particular, Vimentin positivity has been regarded as the major hallmark not only for RCC but also for differentiating them from their benign counterparts, the oncocytomas. Since Hes et al. [8] reported Vimentin positivity in 73% of all tested oncocytomas, this diagnostic approach has been questionable, while its underlying mechanism has been elusive.

From our results, we claim that by using an antibody against the unique C-terminal sequence of Vim3 “real” oncocytomas can be unequivocally identified.

The exact nature and mechanism of the “Vimentin positive oncocytomas” require further clarification. Our results indicate that these tumors have to be classified as an eosinophilic variant of clear cell RCCs. Since their morphologic appearance on an H&E slide seems identical to a “true” oncocytoma, we performed an immune fluorescence for Vim3 (Figure 5). This resulted in a clearly Vim3 negative appearance of these tumors regarded as “true” oncocytomas.

However, the importance of this study for routine pathologic diagnoses with respect to the mystery of “Vimentin positive oncocytomas” advocates our current explanation.

To date, an intracellular role of Vim3 has not been defined while an intracellular role of the full length Vimentin molecule has been described in the literature as an anchoring molecule for the nucleus [16]. Knowing the interaction of its full length counterpart, one may speculate about Vim3's intracellular importance. Since the N-terminal domain and the rod domain have not changed, binding partners such as ankyrin [17] and interactions with plectin [18] should still be possible. In contrast, the missing tail and the unique amino acids of its C-terminal ending may result in differences in the C-terminal interaction. Currently, the tail-domain has been reported to be the binding and interactive site for F-actin [19] and lamin B [17]. However, since the major part of the C-terminus is absent in Vim3 and the exact interaction sites for both molecules are presently unknown, further investigations have to be conducted in order to fully elucidate potential interaction, or its absence, between Vim3 and other structural binding partners. This protein differentiates benign oncocytoma from malignant RCC variants, especially the eosinophilic RCC variant, which mimics oncocytoma by immune histology and immune fluorescence.

**Figure 6:**
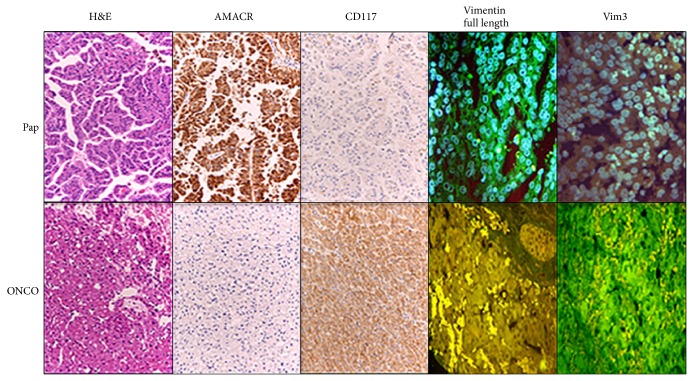
Collision tumor, with two different tumor subtypes. Top row: papillary RCC; bottom row: preliminary diagnosis, oncocytoma. H&E staining and immunohistological staining for AMACR and CD117, Vimentin FL, and Vim3 were performed. Questionable was the second tumor type unexpectedly found in the tissue sample. Immune fluorescence staining for Vimentin FL positive (V9) was positive and the immune fluorescence for Vim3 was negative in the papillary RCC component, indicating the malignant tumor subtype, whereas the second one is an oncocytoma (Vim3 positive).

To strengthen our interpretation, we applied the Vim3 antibody to a collision tumor (Figure 6), in which we identified its metastatic component as belonging to the papillary differentiation being Vim3 negative, while the other part was identified as an oncocytoma, based on its Vim3 positivity. CD117 expression is a hallmark in differentiating oncocytoma and chromophobe RCC [20]. In case of the examined collision tumor, CD117 positivity could be a problem, since the chromophobe RCC is a malignant tumor and the oncocytoma is a benign one, so the differentiation of the metastatic component of the collision tumor is still questionable (Figure 6). The further usage of an alpha-methyl CoA racemase (AMACR) antibody can be used as well for distinction between chromophobe RCC and oncocytoma [21]. AMACR positivity is seen in papillary RCC [22] and can be used as marker between primary and metastatic RCC [21].

Figure 6 shows the two tumor types, and a clear differentiation between the RCC subtypes, namely, the papillary part of the tumor and the benign part, was possible due to the usage of our Vim3 antibody.

In conclusion, we present here a unique Vimentin isoform, Vim3, as a differential marker between malignant RCCs and oncocytoma. We strongly believe that this clear differentiation between the benign and malignant kidney tumor types will be essential in the future for patients' therapy as well as operative planning, follow-up therapy, and patients' survival.

## Figures and Tables

**Table 1:** Tumor types and patient number.

Patient number	Diagnosis
1–6	Normal kidney control
7–22	Oncocytoma
23–33	Chromophobe RCC
34–44	Papillary RCC
45–54	RCC
55–60	Eosinophilic RCC

**Table 2:** Primers.

Gene	Sequence	Annealing temp.	Cycles
*β*-actin	Forw. 5′-TTGGCAATGAGCGGTTCCGCTG-3′ Rev. 5′-TACACGTGTTTGCGGATGTCCAC-3′	55°C	40x

Vimentin, full length	Forw. 5′-GAGAACTTTGCCGTTGAAGC-3′ Rev. 5′-TCCAGCAGCTTCCTGTAGGTG-3′	55°C	40x

Vim3	Forw. 5′-GAGAACTTTGCCGTTGAAGC-3′ Rev. 5′-GAAATAAAATGCTTACCCCTCAG-3′	55°C	40x
